# Measuring Physical Inactivity: Do Current Measures Provide an Accurate View of “Sedentary” Video Game Time?

**DOI:** 10.1155/2014/287013

**Published:** 2014-06-04

**Authors:** Simon Fullerton, Anne W. Taylor, Eleonora Dal Grande, Narelle Berry

**Affiliations:** ^1^Population Research and Outcome Studies, Department of Medicine, University of Adelaide, Level 3, 122 Frome Street, Adelaide, SA 5000, Australia; ^2^Division of Health Sciences, University of South Australia, Adelaide, SA 5000, Australia

## Abstract

*Background*. Measures of screen time are often used to assess sedentary behaviour. Participation in activity-based video games (exergames) can contribute to estimates of screen time, as current practices of measuring it do not consider the growing evidence that playing exergames can provide light to moderate levels of physical activity. This study aimed to determine what proportion of time spent playing video games was actually spent playing exergames. *Methods*. Data were collected via a cross-sectional telephone survey in South Australia. Participants aged 18 years and above (*n* = 2026) were asked about their video game habits, as well as demographic and socioeconomic factors. In cases where children were in the household, the video game habits of a randomly selected child were also questioned. *Results*. Overall, 31.3% of adults and 79.9% of children spend at least some time playing video games. Of these, 24.1% of adults and 42.1% of children play exergames, with these types of games accounting for a third of all time that adults spend playing video games and nearly 20% of children's video game time. *Conclusions*. A substantial proportion of time that would usually be classified as “sedentary” may actually be spent participating in light to moderate physical activity.

## 1. Introduction

Obtaining an accurate view of factors that can affect health and wellbeing is of vital importance. In order to implement strategies to improve overall health through better lifestyle choices, it is necessary to first have a clear picture of the types of behaviour that represent a genuine threat to health, while at the same time identifying those in the community who are most likely to develop such types of behaviour. If data gathered on these types of behaviour is not being interpreted correctly, the efficacy of health promotion strategies may be compromised.

Physical inactivity is increasingly being viewed as one of the most serious public health problems in the developed countries today [1]. Not only does it contribute heavily to the development of many other risk factors (e.g., obesity and high blood pressure), but it can itself be attributed to a large proportion of noncommunicable deaths. Long-term physical inactivity contributes significantly to the secondary aging of various metabolic systems and reduces average life expectancy [2]. It is estimated to cause around 21–25% of breast and colon cancer burden, 27% of diabetes, and about 30% of ischaemic heart disease burden [3]. It was found to be responsible for 1 in 10 deaths in the United States [4], and in a recent large study of Australian adults it was consistently associated with all-cause mortality across a number of different demographic and behavioural factors, including physical activity itself [5]. In Australia, about 16,000 people die prematurely every year because they are not active enough. Lives are at risk, and so too is the economy with the cost of physical inactivity in Australia estimated at $14 billion dollars annually [6].

Current recommendations in Australia suggest that adults should participate in at least 30 minutes of moderate to vigorous physical activity each day [7] and that children should participate in at least 60 minutes [8]. However, recent research indicates that, irrespective of meeting physical activity guidelines, those who spend a significant proportion of their day sedentary are still susceptible to a number of adverse health outcomes such as increased waist circumference, blood pressure and blood glucose, and lipid profiles [9]. It is becoming clearer that not only do we need to encourage people, particularly young people, to get involved in traditional forms of physical activity, but we also need to encourage people to engage in more active forms of entertainment in their leisure time, rather than the more sedentary types of behaviour.

Screen time can account for a large proportion of the time awake spent being physically inactive [10] and includes any activity in which a person is viewing images on a screen, whether it is a television, a computer screen, or a hand-held video game device. However, the differing nature of the various types of screen-based activities may make the dynamics of this relationship ambiguous. There is growing evidence that not all screen-based activities are created equal in terms of factors like energy expenditure [11–14], and so they should not be treated as such when attempting to determine an accurate picture of levels of physical inactivity in the community.

The emergence and growing popularity of “exergames” (video games in which people are required to be physically active in order to play them) in recent years requires us to be a little more specific when determining levels of sedentary time and physical activity. Many of these games require extended periods of moderate levels of activity, similar to walking in relation to energy expenditure [15–17], and currently this is not taken into consideration when calculating time spent being either sedentary or physically active.

The aim of this study was to determine what proportion of time spent playing video games was actually spent playing exergames. Using this, it would be possible to calculate estimates within other data sources as to the amount of video game time that involves a moderate degree of physical activity. This would allow us to understand the degree to which current estimates of physical activity and sedentary behaviour in the community are not fully representative.

## 2. Materials and Methods

### 2.1. Sample

In order to assess the proportion of video game time spent playing exergames, questions were developed and contributed to the May/June 2010 South Australian Health Monitor survey, conducted by Population Research and Outcome Studies (PROS) within the Discipline of Medicine, the University of Adelaide. Health Monitor is a user-pays telephone survey system that conducts large representative surveys of 2000 South Australian households two to three times a year.

Telephone numbers were selected randomly from the electronic white pages (EWP). A letter was sent to each selected household introducing the survey. Only one interview was conducted per household. Where more than one person aged 18 or over resided in the household, the respondent was the person who was last to have their birthday. This was a nonreplacement sample, and up to 10 callbacks were made, if needed, to households to interview the selected person. Respondents were informed that participation in the survey was voluntary, that they could choose to not answer any questions they did not want to, and that they were free to end the interview at any time. Informed consent was obtained from the participant before any survey questions were asked. The May/June 2010 Health Monitor received ethics approval from the Human Research Ethics Committee, SA Health (HREC Protocol number 356/03/2013).

The survey was administered using a CATI (computer-aided telephone interviewing) system whereby respondents' answers were entered directly into the computer by the interviewer. From the 4900 households selected, 2026 interviews were conducted with a participation rate of 64.6% and a response rate of 59.2%. All interviews were conducted by professional health interviewers from 3/5/2010 to 10/6/2010. Telephone calls were made between 10:00 a.m. and 8:30 p.m., seven days a week.

### 2.2. Video Game and Exergame Time

Respondents were first asked how much time they spent playing video games in general. They were then provided with a definition of the term “exergames” (“*exergames is the term used to describe video games that incorporate physical activity as part of their gameplay*”) and were then asked how much time they spent playing those types of games. The data pertaining to children presented below were collected through the adult respondent. Respondents that indicated that there was a child between 5 and 17 years in the household were asked about the age, sex, and video game/exergame habits of the last child to have a birthday in that age range.

### 2.3. Sociodemographic Items

Demographic questions included in the survey were on age, sex, area of residence, dwelling status, country of birth, education level, marital status, gross annual household income, work status, and number of people in the household.

### 2.4. Statistical Analyses

Univariable analyses were performed on the data gathered using SPSS Version 18.0. Data are weighted by area (metropolitan/country), age, gender, and probability of selection in the household to the most recent South Australian population data so that the results are representative of the South Australian population.

## 3. Results and Discussion

Overall, 2026 adult respondents were asked about their gaming habits (specifically, how many hours per day or per week they spent playing them), and of these 537 gave additional information regarding the gaming habits of a child in the household aged between 5 and 17 years. The demographic characteristics of the respondents are presented in Table 1, while the age and sex distribution of the children for whom data regarding video game habits was collected is displayed in Table 2. The mean age of the adult respondents was 47.6 (SD 18.5), while the mean age of the children was 11.0 (SD 3.8).

Table 3 presents the proportion of adults and children that spend any time playing video games and the average amount of time spent playing them.  Table 4 highlights the proportion of these respondents that spend at least some of this time playing exergames and presents the average amount of time spent playing these types of games per day. The average amount of time spent playing video games per day was 15 minutes (SD 38.9) for adults and 40 minutes (SD 53.9) for children. Of those that spent any time playing video games, 24.1% of adults and 42.1% of children reported spending at least some of that time playing exergames. The average amount of time spent playing exergames per day was 5 minutes (SD 13.1) for adults and 8 minutes (SD 14.7) for children.

Based on the means given above, exergames accounted for 19.9% of the total time that children spend playing video games; this equates to 11.9 minutes per hour. Similarly, for adults, exergames accounted for 33.6% of the total time spent playing video games or 20.2 minutes per hour. Results indicate that a quarter of all adults and nearly half of all children that play video games spend at least some of that time playing exergames. On average, it accounts for a third of adult video game time and 20% of children's. The average amount of time per week spent playing exergames was 35 minutes for adults and an hour for children.

## 4. Conclusions

This study highlights that a substantial proportion of video game time is spent playing games that require a light to moderate amount of physical activity. It could be argued that the standard practice of asking people how much time they spend engaged in any kind of screen activity should be amended to include clarification of what proportion of that time is spent playing exergames.

There is a growing body of the literature to support the position that exergames can have a positive effect on the health and wellbeing of children and adolescents that play them. It has been observed to increase heart rate [18, 19], caloric expenditure [20–22], and weight loss, particularly in the overweight and obese youth [23, 24]. There has also been evidence to suggest that participation in exergames can increase the amount of other types of physical activity that children undertake, while decreasing the total amount of time spent playing video games overall [25].

The strengths of this study include a sound methodology that enables confidence in the data gathered as being representative of the South Australian community. However, it is acknowledged that the method of using proxy respondents to gather data on children's gaming habits is less than ideal. Directly surveying the children themselves or better yet monitoring their gaming habits for a period of time would yield more representative data and increase confidence in the relative exergame time proportions.

To the best of our knowledge, no other population-based research has been undertaken to determine what proportion of screen-based time is spent playing exergames. It remains to be seen if the association between screen time and undesirable health outcomes would hold constant if the distinction between exergames and other screen-based activities was factored into calculations. Further research is needed in order to assess whether those who spend their leisure time playing exergames display improved health outcomes compared to those who engage in other, more sedentary, screen-based activities. This can only be achieved if the accuracy in which we measure sedentary behaviour is improved [26].

Historically, campaigns to promote physical activity and also the processes that measure their success have focused almost exclusively on encouraging people to participate in the more traditional types of activities (e.g., group sports, walking, etc.). While these interventions are, and will continue to be, very important with regard to improving health outcomes for people, current research suggests the necessity to widen the focus of such campaigns and processes to include people's leisure time activities. As previously stated, it is becoming clear that the time people spend engaged in sedentary behaviour is a major contributor to poor health outcomes, regardless of their level of activity at other times. Encouraging them to choose more active types of behaviour in their leisure time could be an important step towards dealing with the problem of physical inactivity.

## Figures and Tables

**Table 1:** Demographic characteristics of respondents.

	*n*	%	(95% CI)
Sex			
Male	990	48.9	(46.7–51.0)
Female	1036	51.1	(49.0–53.3)
Age of respondent			
18 to 24	250	12.3	(11.0–13.8)
25 to 34	329	16.2	(14.7–17.9)
35 to 44	366	18.1	(16.5–19.8)
45 to 54	369	18.2	(16.6–19.9)
55 to 64	314	15.5	(14.0–17.1)
65 to 74	200	9.9	(8.6–11.2)
75 and over	199	9.8	(8.6–11.2)
Area			
Metropolitan	1446	71.4	(69.4–73.3)
Country	580	28.6	(26.7–30.6)
Income			
Up to $20,000	258	12.8	(11.4–14.3)
$20,001–$40,000	310	15.3	(13.8–16.9)
$40,001–$60,000	291	14.4	(12.9–16.0)
$60,001–$80,000	243	12.0	(10.7–13.5)
$80,001–$100,000	221	10.9	(9.6–12.3)
More than $100,000	425	21.0	(19.3–22.8)
Not stated/refused/do not know	277	13.7	(12.3–15.2)
Marital status			
Married/living with a partner	1376	67.9	(65.8–69.9)
Separated/divorced	144	7.1	(6.1–8.3)
Widowed	120	5.9	(5.0–7.1)
Never married	382	18.8	(17.2–20.6)
Education			
No schooling to secondary	932	46.1	(43.9–48.2)
Trade/certificate/diploma	630	31.1	(29.1–33.1)
Degree or higher	460	22.8	(21.0–24.6)
Country of birth			
Australia	1573	77.6	(75.8–79.4)
UK/Ireland	199	9.8	(8.6–11.2)
Other	251	12.4	(11.0–13.9)
Household status			
Owned or being purchased by the occupants	1678	83.2	(81.1–84.4)
Renting	290	14.4	(12.8–15.9)
Retirement village	22	1.1	(0.7–1.7)
Other	28	1.4	(1.0–2.0)
Socioeconomic Indexes For Areas (SEIFA) 2006			
The lowest quintile	362	18.0	(16.4–19.7)
Low quintile	427	21.2	(19.5–23.0)
Middle quintile	367	18.2	(16.6–20.0)
High quintile	390	19.4	(17.7–21.2)
The highest quintile	468	23.2	(21.4–25.1)
Overall	**2026**	**100.0**	

The weighting of data can result in rounding discrepancies or totals not adding.

**Table 2:** Demographic characteristics of children in the household for whom data were collected.

	*n*	%	(95% CI)
Age of child			
5 to 7	128	23.8	(20.4–27.6)
8 to 10	130	24.3	(20.8–28.1)
11 to 14	151	28.1	(24.4–32.0)
15 to 17	128	23.9	(20.5–27.6)
Gender of child			
Male	266	49.5	(45.3–53.7)
Female	269	50.1	(45.9–54.3)
Refused	2	0.4	#
Overall	**537**	**100.0**	

The weighting of data can result in rounding discrepancies or totals not adding. ^#^Insufficient cell sizes for statistical tests.

**Table 3:** Time spent playing video games, for adults and children.

	Adults			Children		
	*n*	%	(95% CI)	*n*	%	(95% CI)
Spent any time playing video games						
Yes	633	31.3	(29.3–33.3)	429	79.9	(76.3–83.1)
No	1383	68.3	(66.2–70.2)	103	19.2	(16.1–22.8)
Do not know/refused	10	0.5	(0.3–0.9)	5	0.8	(0.3–2.0)
Hours per day playing video games						
None	1383	68.3	(66.2–70.2)	103	19.2	(16.1–22.8)
Up to half an hour	358	17.7	(16.1–19.4)	229	42.7	(38.6–46.9)
More than half an hour to an hour	145	7.2	(6.1–8.4)	102	19.1	(16.0–22.6)
More than 1 to 2 hours	80	3.9	(3.2–4.9)	59	11.0	(8.7–14.0)
More than 2 hours	50	2.5	(1.9–3.2)	38	7.1	(5.2–9.6)
Do not know/refused	10	0.5	(0.3–0.9)	5	0.5	(0.3–2.0)
Overall	**2026**	**100.0**		**537**	**100.0**	

	*m*	SD	Range	*m*	SD	Range

Mean minutes per day playing video games*	15.10	38.866	0–420	40.65	53.857	0–480

The weighting of data can result in rounding discrepancies or totals not adding. * Including those that did not play video games at all.

**Table 4:** Time spent playing exergames, for adults and children who reported playing video games.

	Adults			Children		
	*n*	%	(95% CI)	*n*	%	(95% CI)
Spent any time playing exergames						
Yes	149	24.1	(20.9–27.7)	181	42.1	(37.5–46.8)
No	467	75.8	(72.3–79.0)	247	57.6	(52.8–62.1)
Do not know/refused	17	2.7	(1.7–4.2)	1	0.3	#
Hours per day playing exergames						
None	467	75.8	(72.3–79.0)	247	57.6	(52.8–62.1)
Up to half an hour	123	19.9	(16.9–23.2)	154	36.0	(31.6–40.6)
More than half an hour to an hour	25	4.1	(2.8–6.0)	23	5.4	(3.6–8.0)
More than 1 to 2 hours	1	0.1	(0.0–0.9)	3	0.7	(0.3–2.1)
Do not know/refused	17	2.7	(1.7–4.2)	1	0.3	#
Overall	**617**	**100.0**		**429**	**100.0**	

	*m*	SD	Range	*m*	SD	Range

Mean minutes per day playing exergames	5.07	13.130	0–120	8.1	14.692	0–120

The weighting of data can result in rounding discrepancies or totals not adding. ^#^Insufficient cell sizes for statistical tests.
